# Supplementary figures and images for: Expression of a gene for an MLX56 defense protein derived from mulberry latex confers strong resistance against a broad range of insect pests on transgenic tomato lines
Source: PLoS One. 2021 Jan 11;16(1):e0239958. doi: 10.1371/journal.pone.0239958 (PMC7799757; doi:10.1371/journal.pone.0239958)

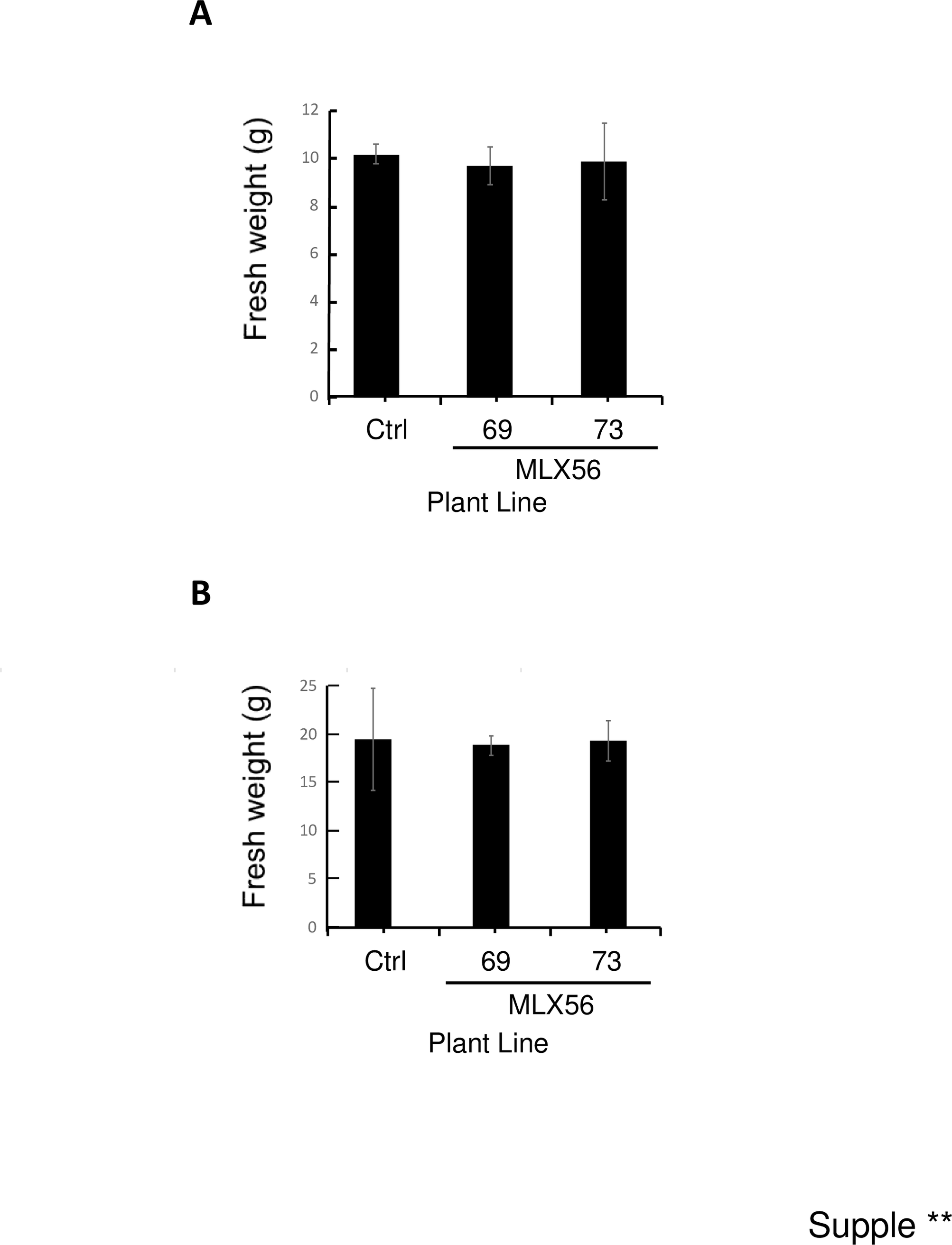

Supplement: S1 Fig — (TIF) [file pone.0239958.s001.tif]

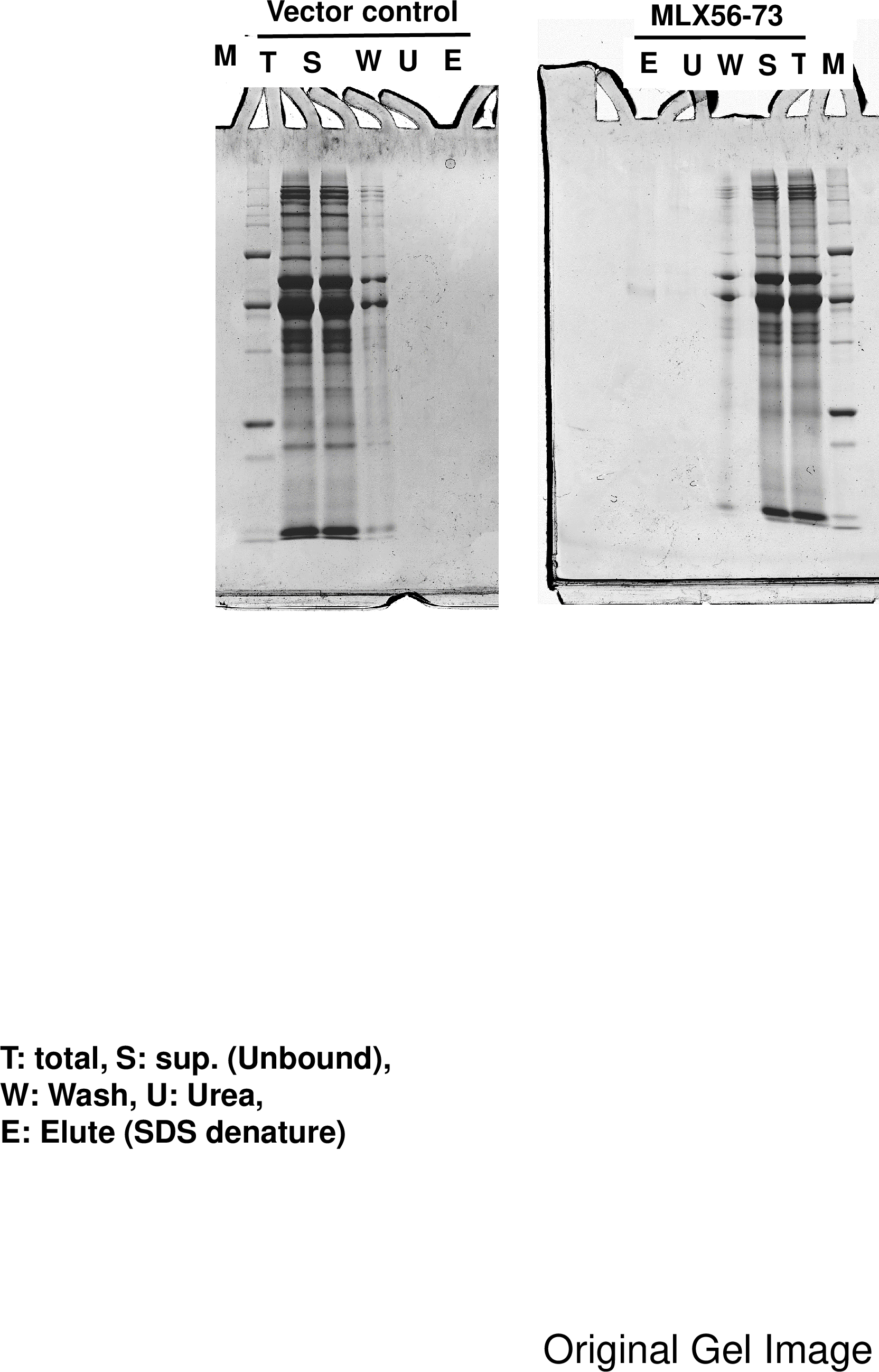

Supplement: S1 Raw Image — (TIF) [file pone.0239958.s005.tif]

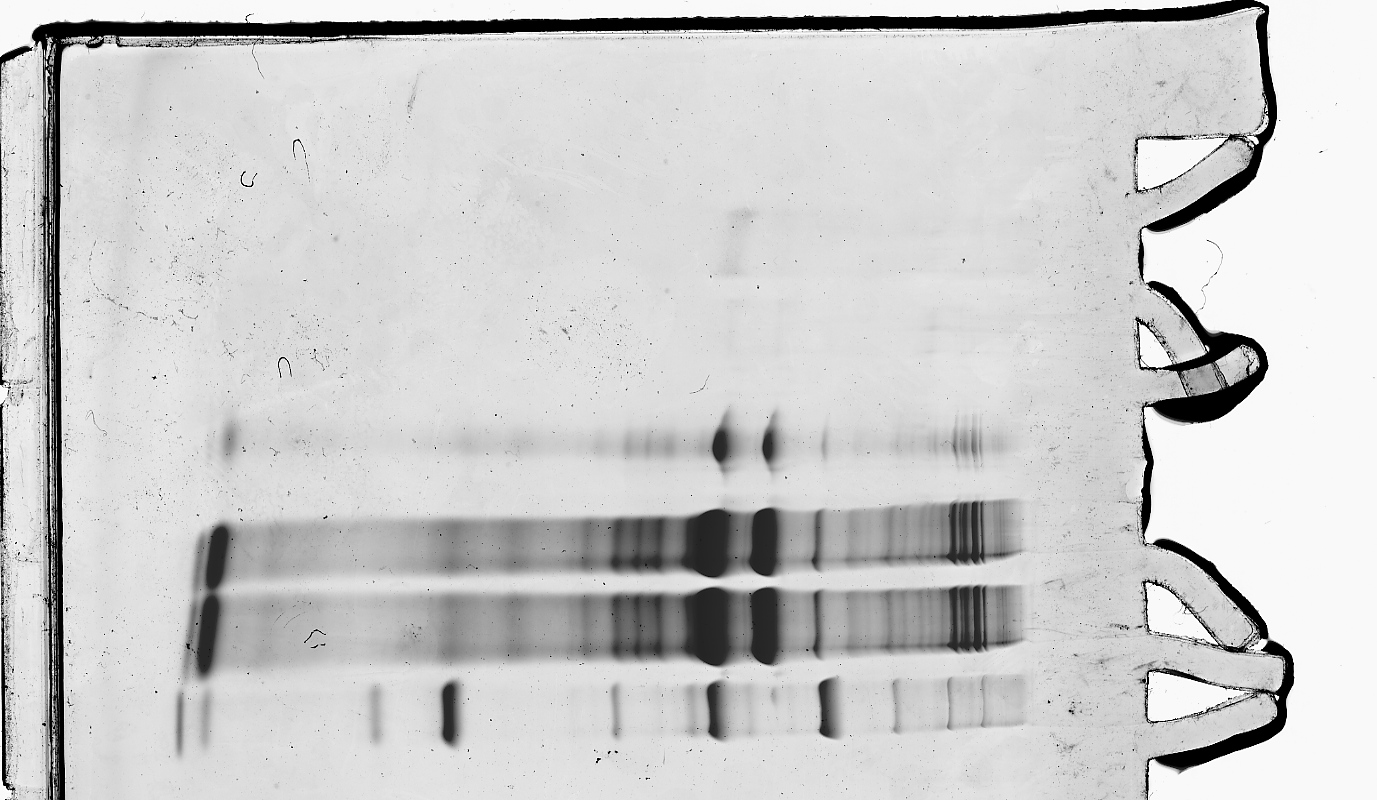

Supplement: S2 Raw Image — (TIF) [file pone.0239958.s006.tif]

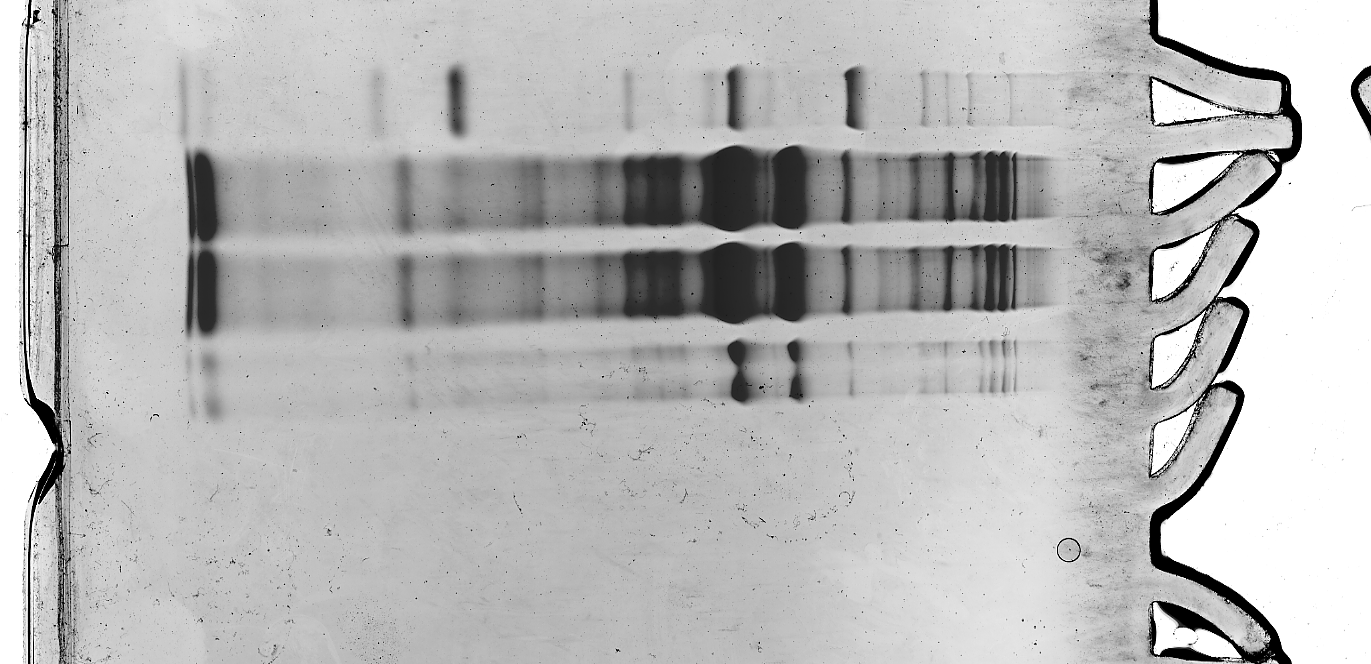

Supplement: S3 Raw Image — (TIF) [file pone.0239958.s007.tif]
